# Antifebrin in the Treatment of Phthisis

**Published:** 1887-05

**Authors:** 


### *ANTIFEBRIN IN THE TREATMENT OF PHTHISIS.*

In an interesting paper in *The Medical Record* for April 16, 1887, Dr. C. H. Cauldwell reported his experience with antifebrin in the treatment of consumption. His conclusions concerning its use are as follows:

1. Antifebrin is the best drug with which to control the chill and fever of phthisis. With it we can at once check these depressing symptoms.

2. It does not produce the unpleasant effects of quinine, salicylic acid, antipyrine, thallin or resorcin.

3. Chills, collapse, or semi-intoxication are not caused by it.

4. In many patients it induces sweating.

5. It diminishes the frequency of the pulse, and usually strengthens the heart's action.

6. Occasionally it produces cyanosis. This happened but twice in thirty cases.

7. It does not interfere with digestion, but on the contrary increases the appetite.

8. Even when the stomach is in an irritable condition, it can be retained.

9. It increases the secretion of urine in the majority of cases.

10. It tends to quiet the nervous system and produce a feeling of "well being" in the patients.

